# Educational preparation of primary care nurse practitioners and outcomes for patients with dementia

**DOI:** 10.1016/j.outlook.2025.102673

**Published:** 2026-01-03

**Authors:** Joshua Porat-Dahlerbruch, Madison Horton, Kyle Featherston, Kathleen Flandrick, Jocelyn Boyd, Lusine Poghosyan, Grant Martsolf

**Affiliations:** a Department of Acute and Tertiary Care, School of Nursing, University of Pittsburgh, Pittsburgh, PA; b Center for Healthcare Delivery Research and Innovation, School of Nursing, Columbia University, New York, NY; c Mailman School of Public Health, Columbia University, New York, NY

**Keywords:** Advanced practice nursing, Aged, Education, Nurse practitioners, Outcomes research

## Abstract

**Background:**

Doctor of Nursing Practice (DNP) enrollment has grown substantially over the past decade. However, there is limited evidence on whether DNP preparation for nurse practitioners (NPs), compared to Master of Science in Nursing (MSN) preparation, leads to improved patient outcomes.

**Purpose:**

To examine differences in emergency department (ED) use and hospitalization among persons living with dementia receiving care from DNP- and MSN-prepared NPs in primary care practices.

**Methods:**

The sample included 603 NPs (509 MSN-prepared, 94 DNP-prepared) and 17,229 patients. Multilevel generalized linear regressions assessed associations between NP educational preparation and odds of ED use and hospitalization.

**Findings:**

Patients receiving care directly from DNP-prepared vs. MSN-prepared NPs did not have lower odds of ED use and hospitalization, but patients receiving care at practices employing DNP-prepared NPs had lower odds of ED use.

**Discussion:**

DNP-prepared NPs in primary care may reduce ED use at the practice level. Further research among other populations is needed.

**Conclusion:**

The DNP may have a positive impact on primary care practices through an educational emphasis on skills such as leadership and quality improvement.

## Background

At the turn of the 21st century, the Institute of Medicine (1996, 2000, 2001, 2003) recommended reforms to primary care to improve quality and reduce system-wide costs. One set of reforms suggested by the Institute of Medicine (2003) included rebuilding provider education programs around five core competencies: (a) provide patient-centered care, (b) work in interdisciplinary teams, (c) employ evidence-based practice, (d) apply quality improvement, and (e) utilize informatics. To incorporate these competencies into graduate-level nursing education, including nurse practitioner (NP) education, the American Association of Colleges of Nursing (2004) recommended transitioning from a Master of Science in Nursing (MSN) to a Doctor of Nursing Practice (DNP) as the standard for entry to practice (McCauley et al., 2020). In alignment with the five core competencies, the American Association of Colleges of Nursing (2006, 2021) advised Schools of Nursing (SONs) to build DNP curricula emphasizing courses in leadership, evidence-based quality improvement, policy, advocacy, translation of research into practice, and technology use.

Since the American Association of Colleges of Nursing’s call to transition to the DNP for entry to practice, DNP enrollment has grown substantially from 70 to 41,831 students across the United States from 2003 through 2023 (American Association of Colleges of Nursing, 2024). The increase in DNP students has required an investment of time and tuition from students and has catalyzed SONs to utilize the scarce nursing faculty workforce to teach in DNP programs (Fang et al., 2024; Halabicky et al., 2024). In return for these investments in DNP programs made by students and SONs, one would expect improved outcomes from enhanced DNP education in the core competencies (e.g., leadership, systems, and evidence-based practice) (Hoyt et al., 2024; Martsolf et al., 2024).

However, few studies have explored whether patients receiving care from DNP-prepared NPs experience improved outcomes (Martsolf et al., 2024; Pappas et al., 2023). The existing evidence indicates that there are no significant patient outcome differences when comparing MSN- and DNP-prepared NPs. As such, researchers have emphasized the need for more robust, standardized data to justify promoting the shift from MSN to DNP preparation (Martsolf et al., 2024; Pappas et al., 2023). There are several reasons for the lack of research on the impact of the DNP on patient outcomes, including historically insufficient sample sizes and the nature of teambased care, leading patients to be linked to other providers in patient outcome databases (Minnick et al., 2019). The only large patient-level outcome study was across six states with about 1,000 primary care NPs; the study found no difference in emergency department (ED) use and hospitalization for patients with chronic conditions receiving care from MSN- and DNP-prepared NPs (Martsolf et al., 2023a). Though this study included a patient sample of older adults with chronic conditions receiving care directly from NPs, and critics of the study have called for additional research on the practice-level outcomes of DNP-prepared NPs (McCurren et al., 2024). That is, McCurren et al. (2024) suggested that the emphasis of DNP curricula on leadership, systems, and evidence-based practice likely contributes directly to improvements in an organization. In turn, improvements in an organization likely would “trickle down” to better direct care outcomes for patients. Therefore, research should examine outcomes linked to the practice and directly to the patients.

There have been several smaller studies with implications for the return-on-investment and added value of the DNP for practices and patients. For example, a mixed-methods study reported that employers do not express a preference for MSN- or DNP-prepared nurses, showing that management may not perceive differences in patient and practice outcomes among MSN- and DNP-prepared NPs (Beeber et al., 2019). A single-university descriptive study of 85 alumni reported that DNP graduates were more likely to be involved in professional organizations, political advocacy, and community service, which are all emphasized more greatly in DNP programs (Inman et al., 2024). Finally, one study conducted in primary care practices found that MSN- and DNP-prepared NPs report non-statistically significant differences in the work environment (Martsolf et al., 2021), indicating no evidence of a benefit of the DNP for practices. The findings of these studies have mixed implications for the value and return-on-investment of the DNP.

The evidence base demonstrating differences in patient outcomes between MSN- and DNP-prepared NPs is scarce and inconclusive. As such, the continued investment in the DNP lacks sufficient empirical support (Martsolf et al., 2024; McCauley et al., 2020). More research is needed to grow the evidence base on the impact of the DNP to understand if patients benefit from the nursing profession’s focus on the DNP (Pappas et al., 2023). To address this gap in the research, we aimed to examine differences in ED use and hospitalization among patients receiving care from MSN- and DNP-prepared NPs in primary care practices.

## Methods

### Study Design

This was a cross-sectional, observational study of persons living with dementia, primary care practices, and NPs across the United States from 2021 through 2023. We focused on persons living with dementia because: (a) it is a large patient population (7 million today, which will grow to 14 million by 2060) (Alzheimer’s Association, 2024; Nandi et al., 2024; United States Census Bureau, 2018, 2023), and (b) dementia can be a key risk factor for other comorbidities and adverse events (e.g., infection, falls, dehydration, malnutrition, cardiovascular disease, and aspiration pneumonia) and when managed poorly in primary care often lead to ED use and hospitalization (Abubakar et al., 2022; Bablok et al., 2021; Fekete et al., 2025; Moo, 2024; Raulin et al., 2022; Sideman et al., 2023; Wingo et al., 2023). Using Medicare claims for persons living with dementia and survey data from NPs providing their care, we examined the link between outcomes of persons living with dementia and NP educational preparation. More details on the survey methods can be found elsewhere (Porat-Dahlerbruch et al., 2025).

### Data Sources

#### Patient Data and Provider Attribution

Using Medicare Fee-For-Service Provider Utilization & Payment Data Physician and Other Supplier Public Use File (Centers for Medicare & Medicaid Services, 2018), we identified beneficiaries aged 65 and older with an Alzheimer’s disease or related dementia diagnosis with at least one ambulatory care visit in either 2018 or 2019. For both years, we attributed beneficiaries to providers billing the highest proportion of evaluation and management visits, given that the visits accounted for at least 30% of the total number of beneficiary primary care visits (Mehrotra et al., 2010). In rare instances of ties (< 1%), we used random attribution (Poghosyan et al., 2025a). This process resulted in persons living with dementia being linked to a unique, main primary care provider.

#### Identifying Patients Receiving Nurse Practitioner Care

We identified persons living with dementia receiving NP care using two different approaches. In our first approach, we included persons living with dementia who had an NP attributed as their main primary care provider (i.e., > 30% of evaluation and management visits) (Martsolf et al., 2023b). In our second approach, we included any person living with dementia attributed to any main primary care provider at a practice employing an NP (Poghosyan et al., 2022b; Poghosyan et al., 2025a; Poghosyan et al., 2025b). Our first approach directly links NPs to the patients they care for. The latter approach aggregates data from all patients to the practice level and is beneficial for two reasons. First, primary care is team-based. That is, patients often receive care from a team of multiple providers, including NPs, in a practice. As such, one would expect to see an effect from the contributions of NPs in a practice, even if NPs may not meet the standard minimum of 30% of evaluation and management visits for attribution (Mehrotra et al., 2010). Second, indirect billing, in which patient visits with NPs are billed to a collaborating physician, occurs in 36% of patient visits (Patel et al., 2022). As such, an NP may be a patient’s main provider, yet NP care may be invisible in administrative claims. Given the strengths of both attribution methods and the lack of evidence on the contribution of education to patients and practices, we chose to use both approaches. In the analysis section, we explain how we approached modeling for both attribution approaches.

#### NP Survey Data

In the parent study, the survey was disseminated to the NPs identified as caring for persons living with dementia in primary care practices. The survey methods involved using the IQVIA OneKey Database to obtain NP name, gender, National Provider Identifier, NP contact information, and practice characteristics (IQVIA Inc., 2020). Using the NP contact information, surveys were sent to all NPs in the sampling frame (*n* = 11,518 NPs across 2,197 practices) via mail and email from November 2021 through September 2023. NPs answered questions about their demographics (age, race, ethnicity, gender, and education), practice work environment (Nurse Practitioner-Primary Care Organizational Climate Questionnaire [NP-PCOCQ]) (Poghosyan et al., 2019), and structural capabilities (Structural Capability Index (Martsolf et al., 2018)). The NP work environment includes scores on four domains of the NP-PCOCQ: NP-Administration Relations, NP-Physician Relations, independent practice and support, and professional visibility domains (Poghosyan et al., 2019). The Structural Capability Index reflects the extent to which practices have adopted resources consistent with high-quality care delivery (Martsolf et al., 2018). The Structural Capability Index captured systems communication (factors advancing timely communication between patients and providers), care coordination (resources to manage patient problem lists, educate patients on self-management, reconcile medications, schedule appointments, communicate with other clinicians, and access social services), chronic disease management registries, and after-hours care (Bilazarian & Martsolf, 2022). We used the NP practice location to identify the Area Deprivation Index (ADI), which uses 17 items from the American Community Survey (domains include income, education, employment, and housing quality) to measure the extent of socioeconomic disadvantage based on practice census block group (Kind & Buckingham, 2018). Finally, we identified any ED use and hospitalization in the Medicare files for patients attributed to the NP survey respondents (or other providers at their practice).

There were 968 NPs who completed the survey. We removed NPs who did not respond to the education questions in the survey. The final analytic sample consisted of 603 NPs across 538 practices. More details on the survey and response rate are reported elsewhere (Porat-Dahlerbruch et al., 2025).

### Variables

#### Independent Variable

The independent variable was the NP’s highest educational preparation, which was sourced from the NP survey data. Each NP was labeled as an MSN- or DNP-prepared NP. Since we were interested in comparing MSN- and DNP-prepared NPs, we excluded NPs reporting that their highest degree was an associate’s, bachelor’s (BSN), or PhD. For the practice-level sample, we labeled each practice as employing MSN- or DNP-prepared NPs (only 16 practices had a mix of NPs with different educational preparation; we dropped these practices from the sample).

#### Dependent Variables

There were two dependent variables: ED use and hospitalization. We sourced these variables from the Medicare claims data. We defined ED use as any visit to the ED in Part B claims for Healthcare Common Procedure Coding System codes (Loyd et al., 2023). We defined hospitalization as any record in the Medicare inpatient claims file with a length of stay of at least 1 day. We counted ED use and hospitalization for each year of data (i.e., 2018 and 2019) (Loyd et al., 2023). As such, a patient may have ED use and/or hospitalization counted in both years of data.

#### Covariates

We included three levels of covariates—patient, NP, and practice characteristics—based on prior studies examining NP care and patient outcomes (Martsolf et al., 2023a; Martsolf et al., 2023b; Poghosyan et al., 2025b; Schlak et al., 2023). We sourced patient characteristics from the Medicare Beneficiary Summary Files data and included age, sex, race, ethnicity, binary variables for 25 of the 27 Chronic Condition Warehouse comorbidities (excluding dementia and Alzheimer’s disease and related dementias) per the ICD-10-CM, and patient ADI (Germack et al., 2022; Martsolf et al., 2023b; Poghosyan et al., 2022a, 2022b). Here, patient sex refers to a binary variable classifying a patient based on biological factors, and NP gender refers to socially constructed roles which the NP respondents self-identified (Kaufman et al., 2023). We sourced NP and practice covariates from the NP survey data. The NP covariates included years in the current practice setting, age, gender, and number of years since receiving initial NP licensure. Practice covariates included practice type (e.g., physician practice, community health center, hospital-based clinic) and practice size (i.e., total count of all providers in an NP’s clinic), scope of practice (full, reduced, or restricted), Structural Capability Index, work environment (the four domains of the NP-PCOCQ), and ADI (national-level percentile).

### Data Analysis

First, we calculated summary statistics and bivariate associations to understand differences among patients receiving care from MSN- and DNP-prepared NPs, their demographics, and the practices where they work. We used chi-squared tests for categorical variables and t-tests for continuous variables. We then ran multivariate, multilevel generalized linear models (with a logistic link function) to determine odds of ED use and hospitalization for each of the two patient-NP attribution methods—i.e., (a) persons living with dementia attributed directly to NPs delivering most care, and (b) persons living with dementia receiving care from any provider at a practice employing NPs. In both regression models, we controlled for patient (comorbidities, age, sex, race, and ADI), NP (age, gender, and years since NP licensure), and practice (practice type, work environment, practice size, Structural Capability Index, and scope of practice) covariates. We accounted for clustering effects by provider by including a random intercept for provider in the multilevel models. In addition, because some patients were in the data in both 2018 and 2019, we included random intercepts for patients in all models to account for multiple observations. Therefore, the final structure of the multilevel model was three-level with multiple observations clustered within patients and patients clustered within providers.

### Ethical Considerations

This study was approved by the Institutional Review Boards of Columbia University Medical Center (IRB-AAAT2116).

## Results

Our attribution approaches yielded two samples. In Supplementary File 1, we include the patient demographics from both the sample of 17,229 patients in the analysis of persons living with dementia directly attributed to NPs in Table S1.1 and the sample of 139,360 patients from the practice-level analysis in Table S1.2. The demographic makeup of the samples is similar. Patients had an average age of 84 years and seven chronic conditions. Most patients were female (approximately 70%) and non-Hispanic White (approximately 85%), with an average ADI of about the 50th percentile. In both 2018 and 2019, practices employing DNP-prepared NPs were located in higher ADIs (i.e., more socioeconomically disadvantaged neighborhoods) (Table S1.1 and S1.2). Patients seeing DNP-prepared NPs (Table S1.1) and receiving care in DNP-employing practices (Table S1.2) tended to have more comorbidities.

In Table 1, we present the NP characteristics. Of the 603 NPs, 509 (84%) held an MSN, and 94 (16%) held a DNP. About 42% of all NPs in the sample were 31 to 45 years old, and almost 50% were 45 to 64 years old. Eleven percent of the NP sample self-reported their gender as male. Almost 70% of the sample received their NP license 3 to 19 years ago. There were no significant differences between MSN- and DNP-prepared NPs in age, gender, and years of experience since licensure.

In Table 2, we report practice characteristics for the 538 practices in the sample. There were 461 practices with MSN- and 77 with DNP-prepared NPs. Most practices were physician-owned, and around half (overall 47.6%; 47.3% of MSN; 49.4% of DNP) of the practices had greater than five providers, with most others employing 3 to 5. In both MSN- and DNP-employing practices, the practice work environment was favorable, with average scores greater than 3 on almost all NP-PCOCQ subscales, indicating majority agreement with positive work environment statements. There were no significant differences between the practices employing MSN- and DNP-prepared NPs.

In Table 3, we present results from logistic regression estimating odds of ED use and hospitalization for patients linked directly to NPs according to the NP’s highest degree (MSN or DNP). In the unadjusted models, there was no significant difference in odds of ED use and hospitalization between patients of MSN- and DNP-prepared NPs (ED use odds ratio (OR): 0.99, *p* = .90; Hospitalization OR: 1.000, *p* = .96). After controlling for patient, NP, and practice characteristics, there were still no statistically significant differences in ED use and hospitalization (ED use OR: 0.95, *p* = .52; hospitalization OR: 0.94, *p* = .44).

In the unadjusted logistic regression of persons living with dementia linked to practices at which NPs work, we found that patients seen at practices employing NPs with DNPs were significantly less likely to use the ED (OR = 0.88, *p* = .03). However, there was no significant difference when analyzing odds of hospitalization (OR = 0.96, *p* = .52) (Table 4). After controlling for patient, NP, and practice characteristics, there remained a statistically significant difference in odds of ED use (OR = 0.87, *p* = .03). Similar to the unadjusted model, there were no significant differences in hospitalization in the adjusted model (OR: 0.98, *p* = .79).

## Discussion

We compared the odds of ED use and hospitalization for persons living with dementia receiving care from MSN- and DNP-prepared NPs. When analyzing data on patients linked directly to NPs providing their care, there were no significant differences in ED use and hospitalization between MSN- and DNP-prepared NPs. When analyzing data from persons living with dementia receiving care from any provider at a practice employing an NP, patients receiving care in practices employing DNP-prepared NPs had lower odds of ED use. There was no significant difference in the odds of hospitalization at the practice level.

While we did not find a relationship between NP education level and outcomes among patients receiving care directly from NPs, we found that practices employing DNP-prepared NPs had significantly lower odds of ED use. It appears that DNP preparation positively contributes to practice-level outcomes. We posit that this practicelevel finding aligns with the added curricular content in DNP programs (Waldrop et al., 2023). Furthermore, contemporary DNP programs focus less on developing clinical skills to provide direct care for patients and more so on leadership, systems, and evidence-based practice (Mainous et al., 2023), potentially improving outcomes of patients receiving care in practices with DNP-prepared NPs. The prior study comparing ED use and hospitalization among patients receiving care from MSN- and DNP-prepared NPs reported results only at the NP level (Martsolf et al., 2023b). Thus, the same effect at the practice level may have been present, though not assessed. We did not find a significant difference in odds of hospitalization among patients in practices employing DNP-prepared NPs, and as such, our hypothesis should be interpreted with caution.

There are other possible explanations for finding lower odds of ED use for persons living with dementia at primary care practices employing DNP-prepared NPs but not in the NP-level models. First, it is possible that practices with better outcomes may tend to hire DNP-prepared NPs. As a counterargument to this point, we attempted to account for some of the potential practice-level characteristics affecting outcomes (practice size, scope of practice, practice type, work environment, and Structural Capability Index). Despite incorporating this relatively robust set of control variables, it is possible that practices employing DNP-prepared NPs are of a higher quality for unobservable reasons. Second, there are five times as many patients in the practice-level sample, creating a possibility that a larger effect size could drive the significant results when examining all patients at a practice vs. those cared for primarily by NPs—139,360 vs. 17,229, respectively. However, this does not seem like the most likely explanation because the odds ratios in the smaller NP-level models were close to 1 (Table 3; unadjusted OR: 0.99, adjusted OR: 0.95), and if the sample size was driving the results, we would expect a lower odds ratio with an insignificant *p* value. Third, it may simply be that DNP programs are not providing advanced clinical education for NP students. Although early proponents envisioned the DNP as a pathway to advanced clinical practice (McCauley et al., 2020), most programs instead emphasize quality improvement and systems-level competencies, which typically benefit the organization. What we are observing, therefore, may be the natural result of the DNP curricular design (Martsolf & Sochalski, 2019; Mundinger & Carter, 2019).

There are several possible explanations for the insignificant difference in odds of hospitalization among patients receiving care at practices employing MSN- and DNP-prepared NPs. First, primary care quality is thought to be associated less with hospitalization than ED use—unlike hospitalizations, ED visits occur earlier in the continuum of health deterioration, making them an earlier warning signal of primary care quality weaknesses (Commonwealth Fund, 2000; O’Malley et al., 2005; Tang et al., 2010). Often, patients will use the ED when primary care is less accessible or of lower quality, while hospitalizations occur after ED use (Currie & Zhang, 2025; Pinchbeck, 2019). As such, the incidence of ED use is more sensitive to primary care quality than hospitalizations. DNP-prepared NPs, moreover, receive formal education about practice management, which often includes implementing models of primary care emphasizing early and proactive management of conditions potentially leading to ED use (Mainous et al., 2023). DNP education, consequently, may lead directly to reductions in odds of ED use, though not necessarily translate to reductions in odds of hospitalization. Also, persons living with dementia tend to be older, have more comorbidities, and require hospitalization more frequently (Lanctot et al., 2024). Accordingly, we would not expect to observe an effect of primary care NP degree in practice or provider levels because hospitalization in these cases occured independent of primary care quality. Of note, in a supplemental analysis, we analyzed ambulatory care sensitive ED use and hospitalizations (preventable with appropriate primary care), and the pattern of results was similar; the odds of ED use were lower in DNP-prepared practices (albeit marginally significant when factoring in all covariates), and odds of hospitalization were not significant (Supplementary Material 2).

### Summary of Implications for National Nursing Education Policy

This is the first nationwide study to identify a significant difference in practice outcomes linked to DNP education. While there is no significance in odds of ED use and hospitalization when examining direct patient care provided by DNP-prepared NPs, odds of ED use were significantly lower at the practice level. As such, this is the first large-scale study to provide evidence supporting the argument that there is a return on investment for primary care practices serving persons living with dementia that employ DNP-prepared NPs. A return on investment for practices could be a reason for continued investment of student time/tuition and SON faculty time in DNP programs. Nevertheless, our argument about return on investment is conceptual—we did not perform a formal return on investment analysis. Future research should study the return on investment of the DNP, especially given the continued growth in DNP enrollments nationwide (American Association of Colleges of Nursing, 2024). Further, this is a single study with a single patient population. More large-scale research on the impact of the MSN vs. DNP at the practice level among other populations (e.g., patients with chronic conditions such as heart failure and NPs in specialty outpatient or inpatient settings) is needed to confirm or refute our findings. Further, qualitative research may help understand why the DNP impacts outcomes at the practice level.

If future studies reinforce our findings, the American Association of Colleges of Nursing may consider two suggestions. First, clarifying that the DNP results in improved practice-level outcomes may inform employers about how DNP-prepared NPs can utilize their advanced education in practice. Second, if the goal of the DNP is to improve direct care outcomes, the American Association of Colleges of Nursing may consider adding curricular content (i.e., competencies) in advanced diagnostic and treatment skills with clinical hours. It may be prudent for the American Association of Colleges of Nursing to sponsor research on outcomes linked to the DNP.

### Limitations

This study had several limitations. First, this was a cross-sectional study, and therefore, we cannot determine causality. Second, this study focused on patients with dementia. Results may differ with other patient populations. Third, for the provider-level analysis, we only included patients directly linked to NPs in the Medicare claims data. If a physician bills Medicare for a patient receiving care from an NP, the patient was not included in the sample. It is known that indirect billing occurs in 36% of ambulatory care visits with NPs (Patel et al., 2022). The impact of indirect billing on patients included in the direct provider-level sample is unknown—the characteristics of these patients have yet to be reported in the literature. It is known, however, that indirect billing is more common in states with a more restricted scope of practice (Patel et al., 2022), and we controlled for scope of practice in the analysis. Fourth, the practice-level analysis was based on the educational preparation level only of the NPs who responded to the survey. It is possible, for example, that some practices that we characterized as “MSN-employing practices” also employed a DNP-prepared NP. The effect of having both MSN- and DNP-prepared NPs in a practice ought to be studied further, as there was not a large enough sample of practices with multiple survey respondents in our data to evaluate whether the practice-level effect is due to employing a single DNP-prepared NP or exclusively DNP-prepared NPs. Finally, we did not explore whether the pathway to obtain a DNP (MSN to DNP or BSN to DNP) impacts our findings because the data were not collected in the survey; future work should explore if the pathway to the DNP affects the relationship between degree level and outcomes.

## Conclusion

While we found no significant differences in patient outcomes between MSN- and DNP-prepared NPs at the individual NP level, we found lower odds of ED use for patients at practices employing DNP-prepared NPs. As such, the DNP may have a positive impact on organizational outcomes through an additional educational emphasis on skills, such as leadership and quality improvement, that positively impact practices. Further large-scale research on differences in outcomes between practices employing DNP- and MSN-prepared NPs is needed across a broader range of patient populations to elucidate whether the DNP is producing a return on investment for practices, patients, and the health care system.

## Supplementary Material

Supplementary File 2

Supplementary File 1

## Figures and Tables

**Table 1:** Nurse Practitioner Characteristics

	Overall *N* = 603	MSN *N* = 509	DNP *N* = 94	*p* Value
*Age, n (%)*				.64
< 30 years	18 (3.0)	17 (3.3)	1 (1.1)	
31–45 years	255 (42.3)	214 (42.0)	41 (43.6)	
45–55 years	153 (25.4)	133 (26.1)	20 (21.3)	
55–65 years	135 (22.4)	111 (21.8)	24 (25.5)	
65+ years	28 (4.6)	22 (4.3)	6 (6.4)	
Unknown	14 (2.3)	12 (2.4)	2 (2.1)	
*Gender, n (%)*				.93
Male	69 (11.4)	59 (11.6)	10 (10.6)	
Female	532 (88.2)	448 (88.0)	84 (89.4)	
Other/unknown	2 (0.3)	2 (0.4)	0 (0.0)	
*Years since receiving initial NP license, n (%)*				.66
0–2 years	93 (15.4)	81 (15.9)	12 (12.8)	
3–8 years	234 (38.8)	199 (39.1)	35 (37.2)	
9–19 years	179 (29.7)	146 (28.7)	33 (35.1)	
20–39+	93 (15.4)	79 (15.5)	14 (14.9)	
Unknown	4 (0.7)	4 (0.79)	0 (0.0)	

*Note. DNP, Doctor of Nursing Practice; MSN, Master of Science in Nursing; NP, nurse practitioner*.

**Table 2:** Practice Characteristics

Characteristic	Total Practices *N* = 538	MSN-Employing Practice *N* = 461	DNP-Employing Practice *N* = 77	*p* Value
*Practice type, n (%)*				.08
Physician practice	328 (61.0)	284 (61.6)	44 (57.1)	
Community health center	42 (7.8)	33 (7.2)	9 (11.7)	
Hospital-based clinic	81 (15.1)	72 (15.6)	9 (11.7)	
Urgent care clinic	28 (5.2)	24 (5.2)	4 (5.2)	
Nurse-managed health center	15 (2.8)	11 (2.4)	4 (5.2)	
Other	41 (7.6)	37 (7.8)	7 (9.1)	
*NP practice environment, mean (SD)*				
NP-physician relations	3.36 (0.5)	3.35 (0.5)	3.41 (0.5)	.40
Independent practice and support	3.51 (0.4)	3.50 (0.4)	3.56 (0.5)	.29
Professional visibility	3.26 (0.6)	3.25 (0.6)	3.30 (0.7)	.51
NP-administration relations	2.90 (0.7)	2.87 (0.7)	3.03 (0.8)	.11
*Practice size, n (%)*				.18
Solo providers	8 (1.5)	8 (1.7)	0 (0.0)	
1–2 providers	100 (18.6)	91 (19.7)	9 (11.7)	
3–5 providers	174 (32.3)	144 (31.2)	30 (39.0)	
> 5 providers	256 (47.6)	218 (47.3)	38 (49.4)	
*Structural capability index, mean (SD)*	0.59 (.23)	0.59 (.25)	0.59 (.22)	.89
*Scope of practice, n (%)*				.39
Full	121 (22.5)	99 (21.5)	22 (28.6)	
Reduced	190 (35.3)	165 (35.8)	25 (32.5)	
Restricted	227 (42.2)	197 (42.7)	30 (39.0)	

*Note*. DNP, Doctor of Nursing Practice; MSN, Master of Science in Nursing; NP, nurse practitioner.

*Note*. For the practice-level sample, we labeled each practice as a practice employing MSN- or DNP-prepared NPs.

**Table 3:** Odds of Emergency Department Use and Hospitalization for Patients Receiving Care From Nurse Practitioners by Nurse Practitioner Highest Degree (DNP or MSN)

Outcome	Overall *N* = 17,229	2018 MSN *n* = 14,093	2018 DNP *n* = 3,136	Unadjusted OR	Unadjusted *p*-value	Adjusted OR	Adjusted *p* Value
ED use, *n* (%)	7,390	5,987	1,403	0.99	.90	0.95	.52
	42.9%	42.5%	44.7%				
Hospitalization, *n* (%)	6,918	5,551	1,367	1.00	.96	0.94	.44
	40.2%	39.4%	43.6%				

Note. DNP, Doctorate in Nursing Practice; ED, emergency department; MSN, Master of Science in Nursing.

*Note.* The model includes data from 2018 and 2019. Models were adjusted for practice, nurse practitioner, and patient characteristics.

**Table 4:** Odds of Emergency Department Use and Hospitalization for Patients Receiving Care at Practices From Any Provider at Practices Employing Nurse Practitioners by Nurse Practitioner Highest Degree (DNP or MSN)

Outcome	Total Patients N = 139,360	MSN-Employing Practice *n* = 121,730	DNP-Employing Practice *n* = 17,630	Unadjusted OR	Unadjusted *p* Value	Adjusted OR	Adjusted *p* Value
ED visit, *n* (%)	58,424	51,263	7,161	0.88	.03	0.87	.03
	41.9%	42.1%	40.6%				
Hospitalization, *n* (%)	55,465	48,069	7,396	0.96	.52	0.98	.79
	39.8%	39.5%	42.0%				

Note. DNP, Doctorate in Nursing Practice; ED, emergency department; MSN, Master of Science in Nursing.

*Note.* The model includes data from 2018 and 2019. Models were adjusted for practice, nurse practitioner, and patient characteristics.

## Appendix A. Supporting information

Supplementary data associated with this article can be found in the online version at doi:10.1016/j.outlook.2025.102673.

## References

[R1] Abubakar MB, Sanusi KO, Ugusman A, Mohamed W, Kamal H, Ibrahim NH, Khoo CS, & Kumar J (2022). Alzheimer’s disease: An update and insights into pathophysiology. Fronteirs in Aging Neuroscience, 14(1), 1–16. 10.3389/fnagi.2022.742408 PMC9006951 35431894

[R2] Alzheimer’s Association (2024). 2024 Alzheimer’s disease facts and figures. Alzheimer’s & Dementia, 20(5), 3708–3821. 10.1002/alz.13809 PMC11095490 38689398

[R3] American Association of Colleges of Nursing. (2004). AACN position statement on the practice doctorate in nursing. https://www.aacnnursing.org/Portals/0/PDFs/Position-Statements/DNP.pdf.

[R4] American Association of Colleges of Nursing. (2006). The essentials of doctoral education for advanced nursing practice. https://www.aacnnursing.org/.

[R5] American Association of Colleges of Nursing. (2021). The essentials: Core competencies for professional nursing education. https://www.aacnnursing.org/essentials. 10.1016/j.profnurs.2025.02.003 40074377

[R6] American Association of Colleges of Nursing. (2024). New AACN data points to enrollment challenges facing U.S. Schools of Nursing. https://www.aacnnursing.org/news-data/all-news/article/new-aacn-data-points-to-enrollment-challenges-facing-us-schools-of-nursing.

[R7] Bablok I, Binder H, Stelzer D, Kaier K, Graf E, Wangler J, Jansky M, Lohr M, Schulz M, Kocklauner M, Geschke K, Wuttke-Linnemann A, Fellgiebel A, Farin E, & study group D (2021). Primary dementia care based on the individual needs of the patient: study protocol of the cluster randomized controlled trial. BMC Geriatrics, 21(1), 1–14. 10.1186/s12877-021-02114-z 33794789 PMC8012747

[R8] Beeber AS, Palmer C, Waldrop J, Lynn MR, & Jones CB (2019). The role of Doctor of Nursing Practice-prepared nurses in practice settings. Nursing Outlook, 67(4), 354–364. 10.1016/j.outlook.2019.02.006 30898369

[R9] Bilazarian A, & Martsolf G (2022). Primary care practice structural capabilities in health professional shortage areas. American Journal of Managed Care, 28(5), 212–217. 10.37765/ajmc.2022.89142 35546584

[R10] Centers for Medicare & Medicaid Services. (2018). Medicare physician & other practitioners by provider. Retrieved May 16, 2025, from https://data.cms.gov/provider-summary-by-type-of-service/medicare-physician-other-practitioners/medicare-physician-other-practitioners-by-provider/data/2018.

[R11] Commonwealth Fund. (2000). Emergency department use in New York City: A survey of Bronx patients. Retrieved August 11, 2025, from https://www.commonwealthfund.org/publications/issue-briefs/2000/nov/emergency-department-use-new-york-city-survey-bronx-patients. 11665700

[R12] Currie J, & Zhang J (2025). Doing more with less: Predicting primary care provider effectiveness. Review of Economics and Statistics, 107(2), 289–305. 10.1162/rest_a_01290 40689273 PMC12276885

[R13] Fang D, Zangaro GA, & Kesten K (2024). Assessment of nursing faculty retirement projections. Nursing Outlook, 72(2), 1–7. 10.1016/j.outlook.2024.102135 38428062

[R14] Fekete M, Liotta EM, Molnar T, Fulop GA, & Lehoczki A (2025). The role of atrial fibrillation in vascular cognitive impairment and dementia: epidemiology, pathophysiology, and preventive strategies. Geroscience, 47(1), 287–300. 10.1007/s11357-024-01290-1 39138793 PMC11872872

[R15] Germack HD, Harrison J, Poghosyan L, & Martsolf GR (2022). Practice patterns, work environments, and job outcomes of rural and urban primary care nurse practitioners. Medical Care Research and Review, 79(1), 161–170. 10.1177/1077558720974537 33213271

[R16] Halabicky OM, Scott PW, Carpio J, & Porat-Dahlerbruch J (2024). Examining observed and forecasted nursing PhD enrollment and graduation trends in the United States: Implications for the profession. Journal of Professional Nursing, 55(1), 81–89. 10.1016/j.profnurs.2024.09.006 39667894

[R17] Hoyt A, Lucey J, Kelly-Weeder S, & O’Reilly-Jacob M (2024). Nurse practitioners’ degrees and associations with time use, functional autonomy, and job outcomes. Nursing Outlook, 72(4), Article 102193. 10.1016/j.outlook.2024.102193 38788269

[R18] Inman D, Taylor KA, Bernheisel KH, Tavakoli AS, & Ribar AK (2024). Outcomes for MSN and DNP graduates: A descriptive study. Journal for Nurse Practitioners, 20(9), 1–3. 10.1016/j.nurpra.2024.105158

[R19] Institute of Medicine (1996). Primary care: America’s health in a new era. National Academies Press 10.17226/5152 25121221

[R20] Institute of Medicine (2000). To err is human: Building a safer health system. National Academies Press 10.17226/9728 25077248

[R21] Institute of Medicine (2001). Crossing the quality chasm: A new health system for the 21st century. National Academies Press 10.17226/10027 25057539

[R22] Institute of Medicine. (2003). Health professions education: a bridge to quality. 10.17226/10681.

[R23] IQVIA Inc. (2020). OneKey reference assets. https://www.iqvia.com/locations/united-states/solutions/life-sciences/information-solutions/essential-information/onekey-reference-assets.

[R24] Kaufman MR, Eschliman EL, & Karver TS (2023). Differentiating sex and gender in health research to achieve gender equity. Bulletin of the World Health Organization, 101(10), 666–671. 10.2471/BLT.22.289310 37772198 PMC10523819

[R25] Kind AJH, & Buckingham W (2018). Making neighborhood disadvantage metrics accessible: The Neighborhood Atlas. New England Journal of Medicine, 378(1), 2456–2458. 10.1056/NEJMp1802313 29949490 PMC6051533

[R26] Lanctot KL, Hviid Hahn-Pedersen J, Eichinger CS, Freeman C, Clark A, Tarazona LRS, & Cummings J (2024). Burden of illness in people with Alzheimer’s disease: A systematic review of epidemiology, comorbidities and mortality. Journal of Prevention of Alzheimer’s Disease, 11(1), 97–107. 10.14283/jpad.2023.61 PMC10225771 38230722

[R27] Loyd C, Blue K, Turner L, Weber A, Guy A, Zhang Y, Martin RC, Kennedy RE, & Brown C (2023). National norms for hospitalizations due to ambulatory care sensitive conditions among adults in the US. Journal of General Internal Medicine, 38(13), 2953–2959. 10.1007/s11606-023-08161-z 36941421 PMC10027258

[R28] Mainous RO, Dunlap JJ, & Brewer TL (2023). Realizing the DNP as envisioned: Moving toward consistent nomenclature, curricula, and outcomes. Nursing Outlook, 71(3), 1–4. 10.1016/j.outlook.2023.101969 37023671

[R29] Martsolf GR, Ashwood S, Friedberg MW, & Rodriguez HP (2018). Linking structural capabilities and workplace climate in community health centers. 46958018794542 Inquiry, 55. 10.1177/0046958018794542 PMC6120169 30168364

[R30] Martsolf GR, Kandrack R, Ferrara SA, & Poghosyan L (2023a). The impact of the New York Nurse Practitioner Modernization Act on the employment of nurse practitioners in primary care. 469580231171333 Inquiry: The Journal of Health Care Organization, Provision, and Financing, 60. 10.1177/00469580231171333 PMC10161305 37139742

[R31] Martsolf GR, Komadino A, Germack H, Harrison J, & Poghosyan L (2021). Practice environment, independence, and roles among DNP- and MSN-prepared primary care nurse practitioners. Nursing Outlook, 69(6), 953–960. 10.1016/j.outlook.2021.06.008 34446293

[R32] Martsolf GR, Porat-Dahlerbruch J, & Poghosyan L (2024). Response to Drs. McCurren and Trautman. Nursing Outlook, 72(1), 1. 10.1016/j.outlook.2023.102101 38065825

[R33] Martsolf GR, & Sochalski J (2019). The need for advanced clinical education for nurse practitioners continues despite expansion of doctor of nursing practice programs. Policy Politics and Nursing Practice, 20(4), 183–185. 10.1177/1527154419882310 31640458

[R34] Martsolf G, Turi E, Liu J, Chen J, & Poghosyan L (2023b). DNP preparation of primary care nurse practitioners and clinical outcomes for patients with chronic conditions. Nursing Outlook, 71(3), 1–9. 10.1016/j.outlook.2023.101951 PMC10683711 36947974

[R35] McCauley LA, Broome ME, Frazier L, Hayes R, Kurth A, Musil CM, Norman LD, Rideout KH, & Villarruel AM (2020). Doctor of nursing practice (DNP) degree in the United States: Reflecting, readjusting, and getting back on track. Nursing Outlook, 68(4), 494–503. 10.1016/j.outlook.2020.03.008 32561157 PMC7161484

[R36] McCurren C, Trautman D, & Rosseter R (2024). Response to “DNP preparation of primary care nurse practitioners and clinical outcomes of patients with chronic conditions”. Nursing Outlook, 72(1), 1–2. 10.1016/j.outlook.2023.102055 37813800

[R37] Mehrotra A, Adams JL, Thomas JW, & McGlynn EA (2010). The effect of different attribution rules on individual physician cost profiles. Annals of Internal Medicine, 152(10), 649–654. 10.7326/0003-4819-152-10-201005180-00005 20479030 PMC2990946

[R38] Minnick AF, Kleinpell R, & Allison TL (2019). DNPs’ labor participation, activities, and reports of degree contributions. Nurings Outlook, 67(1), 89–100. 10.1016/j.outlook.2018.10.008 30579561

[R39] Moo LR (2024). Longitudinal management of dementia in primary care. JAMA Internal Medicine, 184(5), 459–460. 10.1001/jamainternmed.2023.8513 38436970

[R40] Mundinger MO, & Carter MA (2019). Potential crisis in nurse practitioner preparation in the United States. Policy Politics and Nursing Practice, 20(2), 57–63. 10.1177/1527154419838630 30943837

[R41] Nandi A, Counts N, Broker J, Malik S, Chen S, Han R, Klusty J, Seligman B, Tortorice D, Vigo D, & Bloom DE (2024). Cost of care for Alzheimer’s disease and related dementias in the United States: 2016 to 2060. NPJ Aging, 10(1), 1–8. 10.1038/s41514-024-00136-6 38331952 PMC10853249

[R42] O’Malley AS, Gerland AM, Pham HH, & Berenson RA (2005). Rising pressure: Hospital emergency department as barometers of the health care system. Center for Studying Health System Change, Issue. http://www.hschange.org/CONTENT/799/. 16299951

[R43] Pappas S, Davis KK, Dubree M, Foxx M, Fuchs MA, Hayes R, Manzano W, Mattioni C, & Williams E (2023). Nursing practice leaders’ perspectives on continuing challenges related to the doctor of nursing practice degree. Nursing Outlook, 71(4), 1–10. 10.1016/j.outlook.2023.102002 37481348

[R44] Patel SY, Huskamp HA, Frakt AB, Auerbach DI, Neprash HT, Barnett ML, James HO, & Mehrotra A (2022). Frequency of indirect billing to Medicare for nurse practitioner and physician assistant office visits. Health Affairs, 41(6), 805–813. 10.1377/hlthaff.2021.01968 35666969 PMC9373851

[R45] Pinchbeck EW (2019). Convenient primary care and emergency hospital utilisation. Journal of Health Economics, 68(1), 1–16. 10.1016/j.jhealeco.2019.102242 31605834

[R46] Poghosyan L, Dougherty M, Martsolf GR, Featherston K, Porat-Dahlerbruch J, Borson S, Sadak T, Wang S, & O’Reilly-Jacob M (2025a). Dementia care management in primary care practices: A descriptive study among nurse practitioners. BMC Primary Care, 26(1), 1–10. 10.1186/s12875-025-02855-5 40375125 PMC12080150

[R47] Poghosyan L, Ghaffari A, & Shaffer J (2019). Nurse practitioner primary care organizational climate questionnaire: Item response theory and differential item functioning. Journal of Clinical Nursing, 28(15-16), 2934–2945. 10.1111/jocn.14895 31013392

[R48] Poghosyan L, Kueakomoldej S, Liu J, & Martsolf G (2022a). Advanced practice nurse work environments and job satisfaction and intent to leave: Six-state cross sectional and observational study. Journal of Advanced Nursing, 78(8), 2460–2471. 10.1111/jan.15176 35174905 PMC9283202

[R49] Poghosyan L, Liu J, Chen JL, Flandrick K, McMenamin A, Porat-Dahlerbruch J, Rowell-Cunsolo TL, & Martsolf GR (2025b). Racial disparities in hospitalization and neighborhood deprivation among Medicare beneficiaries. Health Affairs Scholar, 3(2), 1–9. 10.1093/haschl/qxaf010 PMC11803629 39927097

[R50] Poghosyan L, Liu J, Perloff J, D’Aunno T, Cato KD, Friedberg MW, & Martsolf G (2022b). Primary care nurse practitioner work environments and hospitalizations and ED use among chronically ill Medicare beneficiaries. Medical Care, 60(7), 496–503. 10.1097/MLR.0000000000001731 35679173 PMC9202077

[R51] Porat-DahlerbruchJ, HortonM, FlandrickK, FeatherstonK, PoghosyanL, & MartsolfG (2025). Methods and lessons from conducting a national study of primary care nurse practitioner. Journal of the American Association of Nurse Practitioners, 13(6), 1–11. 10.1097/JXX.000000000000113140227280

[R52] Raulin AC, Doss SV, Trottier ZA, Ikezu TC, Bu G, & Liu CC (2022). ApoE in Alzheimer’s disease: Pathophysiology and therapeutic strategies. Molecular Neurodegeneration, 17(1), 1–26. 10.1186/s13024-022-00574-4 36348357 PMC9644639

[R53] Schlak A, Poghosyan L, Rosa WE, Mathew S, Liu J, Martsolf G, Flandrick K, & Chen JL (2023). The impact of primary care practice structural capabilities on nurse practitioner burnout, job satisfaction, and intent to leave. Medical Care, 61(12), 882–889. 10.1097/MLR.0000000000001931 37815323 PMC10695280

[R54] Sideman AB, Ma M, Hernandez de Jesus A, Alagappan C, Razon N, Dohan D, Chodos A, Al-Rousan T, Alving LI, Segal-Gidan F, Rosen H, Rankin KP, Possin KL, & Borson S (2023). Primary care pracitioner perspectives on the role of primary care in dementia diagnosis and care. JAMA Network Open, 6(9), 1–13. 10.1001/jamanetworkopen.2023.36030 PMC10539983 37768660

[R55] Tang N, Stein J, Hsia RY, Maselli RH, & Gonzales R (2010). Trends and characteristics of US emergency department visits, 1997-2007. Journal of the American Medical Association, 304(6), 664–670. 10.1001/jama.2010.1112 20699458 PMC3123697

[R56] United States Census Bureau. (2018). Older people projected to outnumber children for first time in U.S. history. https://www.census.gov/newsroom/press-releases/2018/cb18-41-population-projections.html.

[R57] United States Census Bureau. (2023). The older population: 2020. https://www2.census.gov/library/publications/decennial/2020/census-briefs/c2020br-07.pdf.

[R58] Waldrop J, Reynolds SS, McMillian-Bohler JM, Graton M, & Ledbetter L (2023). Evaluation of DNP program essentials of doctoral nursing education: A scoping review. Journal of Professional Nursing, 46(1), 7–12. 10.1016/j.profnurs.2022.11.009 37188425

[R59] Wingo TS, Liu Y, Gerasimov ES, Vattathil S, Wynne ME, Faundez V, Liu J, Bennett DAA, Seyfried NT, Levey AI, & Wingo AP (2023). A genetic and proteomic approach to understanding neuropsychiatric symptoms in ADRD. Alzheimer’s & Dementia, 19(S18), 1–2. 10.1002/alz.070956

