# Supplementary material for: Educational preparation of primary care nurse practitioners and outcomes for patients with dementia
Source: Nurs Outlook. Author manuscript; Available in PMC 2026 May 28. (PMC13217506; doi:10.1016/j.outlook.2025.102673)
Supplement: Supplementary File 2 [file NIHMS2166815-supplement-Supplementary_File_2.docx]

| Table S1.1 –Patient demographics from the analysis of persons living with dementia attributed to NPs | | | | | | |
| --- | --- | --- | --- | --- | --- | --- |
|  |  |  |  |  |  |  |
| Patient Characteristics | Overall  N = 17,229 | 2018 MSN  N = 7,429 | 2018 DNP  N = 1,590 | 2019 MSN  N = 6,664 | 2019 DNP  N = 1,546 | p-value |
| Age – mean (SD) | 83.5 (8.5) | 83.3 (8.6) | 83.2 (8.4) | 83.7 (8.5) | 83.9 (8.3) | .01 |
| Number of chronic conditions – mean (SD) | 7.1 (2.9) | 6.9 (2.9) | 7.2 (3.0) | 7.1 (2.9) | 7.6 (2.9) | <.001 |
| ADI National Rank – mean (SD) | 52.2 (27.13) | 52.7 (27.5) | 53.2 (25.8) | 51.05 (27.2) | 53.5 (26.4) | <.001 |
| Female sex – n (%) | 11,824 (68.6) | 5,125 (69.0) | 1,053 (66.2) | 4,565 (68.5) | 1,081 (69.9) | .11 |
| Race – n (%) |  |  |  |  |  | <.001 |
| American Indian/Alaska Native | 40 (0.2) | 18 (0.2) | 2 (0.1) | 19 (0.3) | 1 (0.1) |  |
| Asian | 314 (1.8) | 157 (2.1) | 12 (0.8) | 132 (2.0) | 13 (0.8) |  |
| Black | 1,511 (8.8) | 632 (8.5) | 187 (11.8) | 512 (7.7) | 180 (11.6) |  |
| Hispanic | 563 (3.3) | 265 (3.6) | 54 (3.4) | 204 (3.1) | 40 (2.6) |  |
| Non-Hispanic White | 14,633 (84.9) | 6,278 (84.5) | 1,326 (83.4) | 5,727 (85.9) | 1,302 (84.2) |  |
| Other | 103 (0.6) | 51 (0.7) | 2 (0.1) | 47 (0.7) | 3 (0.2) |  |
| Unknown | 65 (0.4) | 28 (0.4) | 7 (0.4) | 23 (0.4) | 7 (0.5) |  |

**SUPPLEMENTARY FILE 1**

*Note: P-values were calculated based on a combined 2018 and 2019 sample. That is, all patients with an MSN-prepared NP provider in 2018 and all patients with an MSN-prepared NP provider in 2019 were combined into one group, and all patients with a DNP-prepared NP provider in 2018 and all patients with a DNP-prepared NP provider in 2019 were combined into one group. Then, these patients linked to MSN-prepared NPs in both years were compared to all patients linked to DNP-prepared NPs in both years.*

| Table S1.2 –Patient demographics from the analysis persons living with dementia receiving care from any provider at a practice employing nurse practitioners | | | | | | | | | | |  |
| --- | --- | --- | --- | --- | --- | --- | --- | --- | --- | --- | --- |
|  | | | | | | | | | | |  |
| Patient Characteristics | Overall  N= 139,360 | | 2018 MSN  N = 64,720 | | 2018 DNP  N = 9,330 | | 2019 MSN  N= 57,010 | | 2019 DNP  N = 8,300 | | p-value |
|  | M | SD | M | SD | M | SD | M | SD | M | SD |  |
| Age | 82.9 | 8.4 | 82.8 | 8.4 | 82.8 | 8.4 | 83.0 | 8.4 | 83.2 | 8.3 | .07 |
| ADI National Rank | 51.0 | 27.5 | 50.7 | 27.6 | 56.7 | 25.9 | 49.8 | 27.6 | 56.0 | 26.2 | <.001 |
| CCW count | 7.1 | 2.9 | 6.9 | 2.9 | 7.1 | 3.0 | 7.3 | 2.9 | 7.3 | 3.0 | <.001 |
|  | N | % | N | % | N | % | N | % | N | % |  |
| Female sex | 89,455 | 64.2 | 41,546 | 64.2 | 5,927 | 63.5 | 36,611 | 64.2 | 5,371 | 64.2 | .10 |
| Race |  |  |  |  |  |  |  |  |  |  | <.001 |
| American Indian/Alaska Native | 478 | 0.3 | 242 | 0.4 | 16 | 0.2 | 209 | 0.4 | 11 | 0.1 |  |
| Asian | 2,402 | 1.7 | 1,129 | 1.7 | 61 | 0.7 | 1,158 | 2.0 | 54 | 0.7 |  |
| Black | 11,618 | 8.3 | 5,109 | 7.9 | 854 | 9.2 | 4,816 | 8.5 | 839 | 10.1 |  |
| Hispanic | 4,339 | 3.1 | 1,851 | 2.9 | 290 | 3.1 | 1,931 | 3.4 | 267 | 3.2 |  |
| Non-Hispanic white | 119,042 | 85.4 | 55,744 | 86.1 | 8,027 | 86.0 | 48,221 | 84.6 | 7,050 | 84.9 |  |
| Other | 848 | 0.6 | 393 | 0.6 | 35 | 0.4 | 390 | 0.7 | 30 | 0.4 |  |
| Unknown | 633 | 0.5 | 252 | 0.4 | 47 | 0.5 | 285 | 0.5 | 49 | 0.6 |  |

*Note: P-values were calculated based on a combined 2018 and 2019 sample. That is, all patients seen at a practice employing MSN-prepared NPs in 2018 and all patients seen at a practice with MSN-prepared NPs in 2019 were combined into one group, and all patients seen at practice employing DNP-prepared NPs in 2018 and all patients seen at practices employing DNP-prepared NPs in 2019 were combined into one group. Then, these patients linked to MSN-prepared NP practices in both years were compared to patients linked to practices employing DNP-prepared NPs in both years.*
