# Supplementary material for: Educational preparation of primary care nurse practitioners and outcomes for patients with dementia
Source: Nurs Outlook. Author manuscript; Available in PMC 2026 May 28. (PMC13217506; doi:10.1016/j.outlook.2025.102673)
Supplement: Supplementary File 1 [file NIHMS2166815-supplement-Supplementary_File_1.docx]

**SUPPLEMENTARY FILE 2: SENSITIVITY ANALYSIS OF AMBULATORY-CARE SENSITIVE OUTCOMES**

**Table S2.1:** Sensitivity Analysis: Odds of Emergency Department Use and Hospitalization for Patients Receiving Care from Nurse Practitioners by Nurse Practitioner Highest Degree (DNP or MSN)

| Outcome | Overall  N=17,229 | 2018 MSN  n=14,093 | 2018 DNP  n=3,136 | Unadjusted OR | Unadjusted p-value | Adjusted OR | Adjusted p-value |
| --- | --- | --- | --- | --- | --- | --- | --- |
| ACS  ED use  n (%) | 4,301 25.0% | 3,478  24.7% | 823  26.2% | 0.98 | 0.81 | 0.92 | 0.43 |
| ACS Hospitalization n (%) | 1,010 5.9% | 801  5.7% | 209  6.7% | 1.11 | 0.78 | 0.97 | 0.85 |

***Note.*** DNP, Doctorate in Nursing Practice; MSN, Master of Science in Nursing; ED, emergency department; ACS: Ambulatory Care-Sensitive. The model includes data from 2018 and 2019. Models were adjusted for practice, nurse practitioner, and patient characteristics.

**Table S2.2:** Sensitivity Analysis: Ambulatory Care-Sensitive Odds of Emergency Department Use and Hospitalization for Patients Receiving Care at Practices from Any Provider at Practices Employing Nurse Practitioners by Nurse Practitioner Highest Degree (DNP or MSN)

| Outcome | Total patients  N=139,360 | MSN-Employing Practice  n=121,730 | DNP-Employing Practice  n=17,630 | Unadjusted OR | Unadjusted p-value | Adjusted OR | Adjusted p-value |
| --- | --- | --- | --- | --- | --- | --- | --- |
| ACS  ED visit  n (%) | 34,584  24.8% | 30,422  25.0% | 4,162  23.6% | 0.90 | .03 | 0.89 | .05 |
| ACS Hospitalization n (%) | 8,806  6.3% | 7,592  6.2% | 1,214  6.9% | 1.11 | .37 | 0.99 | .88 |

***Note.*** DNP, Doctorate in Nursing Practice; MSN, Master of Science in Nursing; ED, emergency department; ACS: Ambulatory Care-Sensitive. The model includes data from 2018 and 2019. Models were adjusted for practice, nurse practitioner, and patient characteristics.
